# Infrastructure support for short food supply chains: the current state of play in England and towards a research agenda

**DOI:** 10.1186/s40100-025-00418-x

**Published:** 2025-11-16

**Authors:** Damian Maye, Matthew Gorton, Aimee Morse, Barbara Tocco, Marie Steytler

**Affiliations:** 1 University of Gloucestershire, Cheltenham, UK; 2 Newcastle University, Newcastle upon Tyne, UK; 3 Corvinus University, Budapest, Hungary

**Keywords:** Short food supply chains, Infrastructure support, Research agenda, England

## Abstract

Drawing on survey, interview, and workshop evidence, this paper reviews the current state of play of short food supply chains (SFSCs) in England, calling for greater focus on infrastructure to support and enable SFSCs to build capacity in agri-food economies. The paper argues that despite a recent burgeoning of research on SFSCs, the role and importance of infrastructure to support SFSC arrangements remain piecemeal and too often ‘backstage’. A survey of 586 farms in England (completed May–August 2023) identifies widespread and greater than anticipated interest in increasing engagement in SFSCs, including amongst larger and crop farms. However, inadequate infrastructure is perceived as a major barrier to market access. Interviews (*n* = 29) (conducted in 2023) and a participatory workshop (February 2024), both involving SFSC operators, advisory organisations, and academics in England, echo survey findings and help understand why reconfiguring infrastructure is essential for transforming national, regional, and local food systems. The paper concludes by outlining priority topics, identified by practitioners, for future academic research. Key steps for a future research agenda include developing a common understanding of infrastructure types and combinations for sectors, alongside wider strategic alliance building with recognition that infrastructure support alone (material, virtual, legal, etc.) will not be sufficient. Bringing infrastructure ‘front stage’ in this more strategic way, we conclude, builds resilience capacity in agri-food economies to enable producers interested in SFSCs to realise positive outcomes.

## Introduction

Short food supply chains (hereafter ‘SFSCs’) have proliferated both as material practices and objects of food systems research in the last two decades. From a practice perspective, SFSCs comprise a plethora of market-based initiatives that in some capacity ‘shorten’ the connection between producers and consumers. Their diversity now extends well beyond rural domains to include operations in urban and peri-urban settings, comprising ‘traditional’, ‘neo-traditional’, and ‘modern’ arrangements (Chiffoleau and Dourian 2020) and broadly categorised as follows (UNIDO 2020): on-farm selling, farmers’ markets, farm shops and box schemes, consumer-driven initiatives, and public and private procurement (hotels, restaurants, catering).

**Fig. 1:**
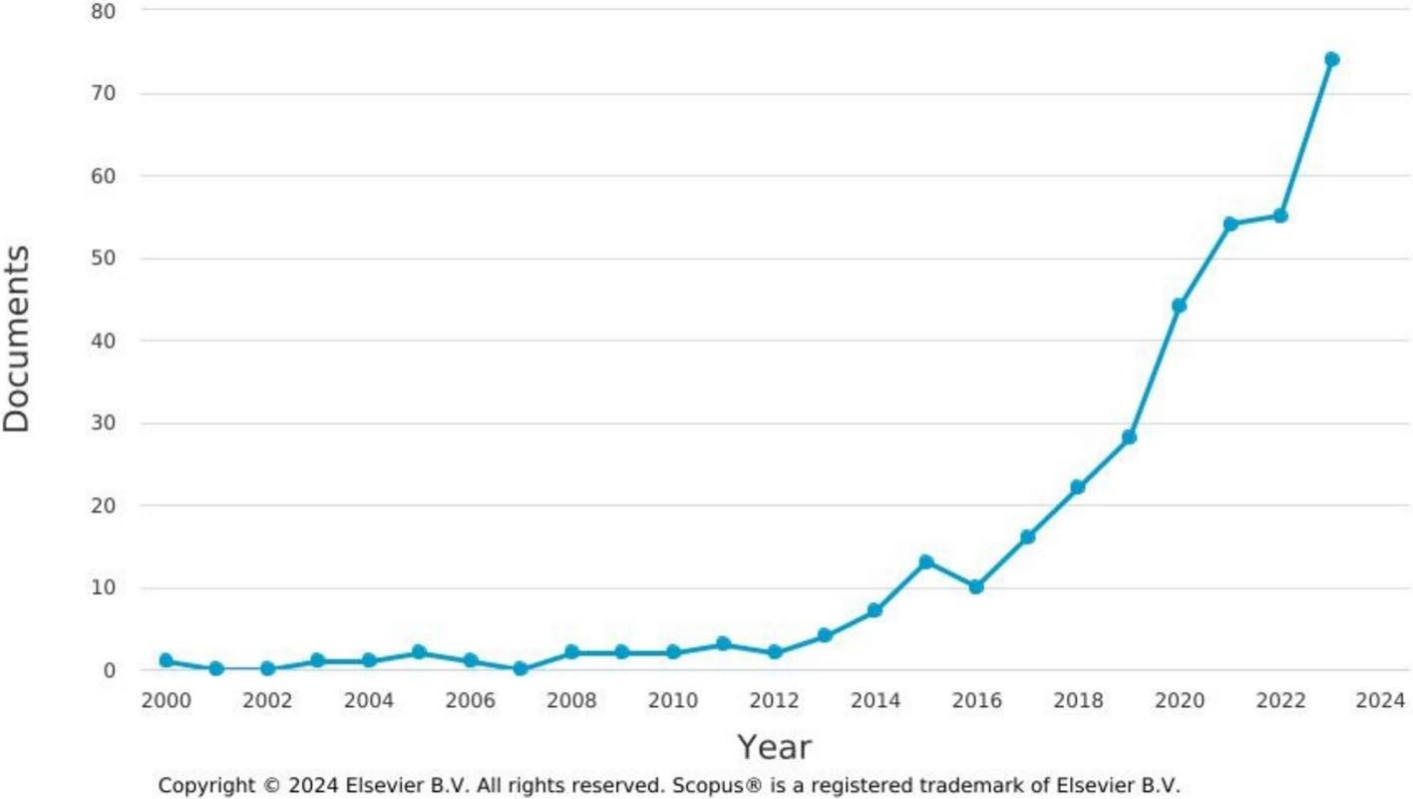
SFSC articles published over time (Source: Scopus). *Note:* search coverage is English language academic studies of SFSCs

The rising popularity of SFSCs amongst consumers and producers is reflected in academic studies, as evidenced through a review of SFSC research in Scopus and Web of Science. Figure 1 summarises social science and related articles using the term ‘short food supply chain’ since Marsden et al. (2000) first introduced the concept. For the first 10 years or so, the concept was relatively marginal, but we see an uptake since then and quite significant interest in the concept in recent years, especially during and after the Covid-19 global pandemic. This trend matches those reported by Jia et al. (2024) and Petruzzelli et al. (2023). As noted by Chiffoleau and Dourian (2020), the adaptability of local food systems during Covid-19 stimulated renewed interest from researchers and policymakers in what these chains might deliver for food systems (see too Baptista et al. 2022).

**Fig. 2:**
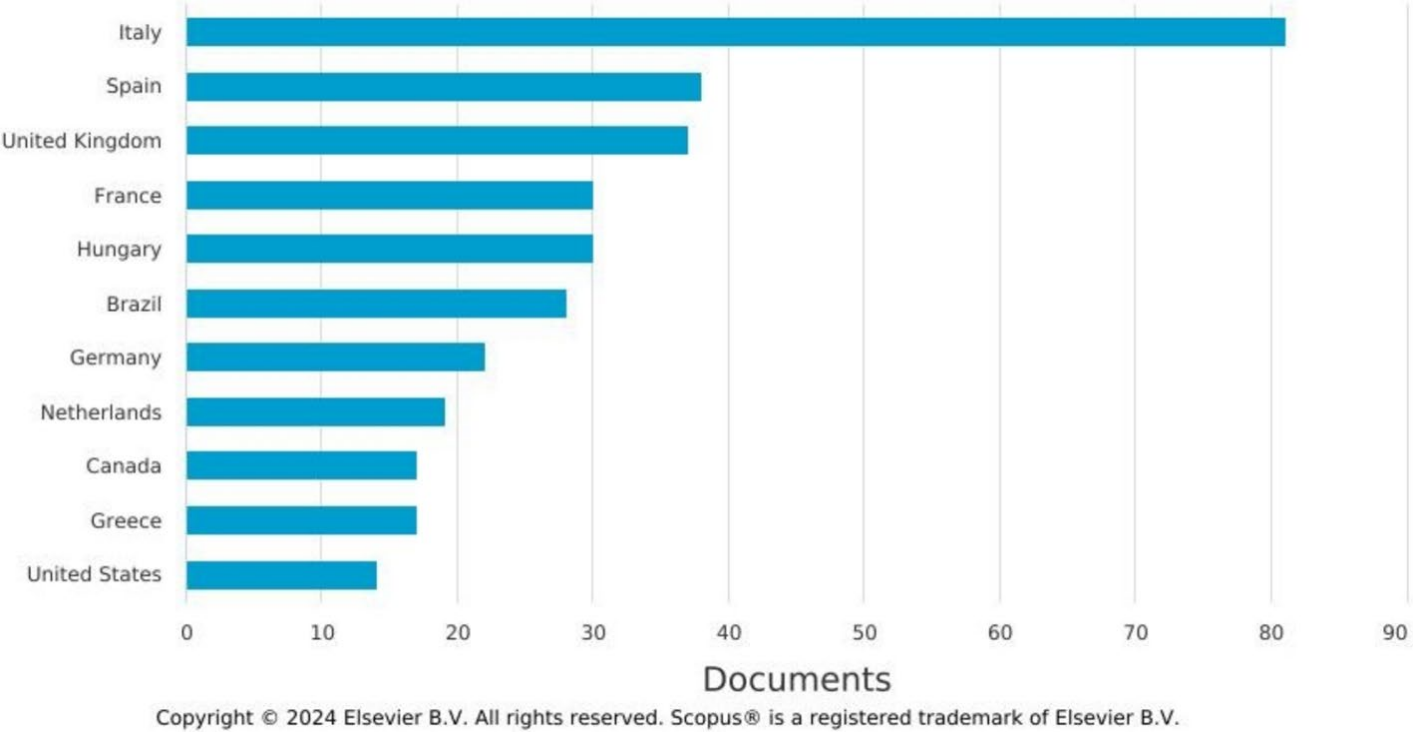
SFSC articles published by country (Source: Scopus). *Note:* search coverage is restricted to English language academic studies of SFSCs

Two other patterns emerge from Scopus and Web of Science that are of interest for this paper. The first relates to *where* SFSC studies emerge. The academic and sociological heritage of the SFSC concept is European rural development scholarship (see van der Ploeg et al. 2000). This is seemingly maintained (Fig. 2). We see, for example, Italy with the highest number of publications followed by Spain, the UK, France, and Hungary. However, the concept is also travelling to, e.g. Brazil, which has seen much debate regarding renewing school meal public food programmes as shorter more localised market arrangements (Schneider et al. 2016); we also see uptake of the concept in Canada and the USA. Jia et al. (2024) also analyse ‘top cited articles’ and ‘most cited countries’, identifying the highest interest in the USA, China, and Canada, and, within Europe, Italy, the UK, and Germany.

The Scopus and Web of Science searches identified also several recent SFSC review articles summarising the *state of the art* (e.g. Baptista et al. 2022; Chiffoleau and Dourian 2020; Evola et al. 2022; Jia et al. 2024; Paciarotti and Torregiani 2021; Petruzzelli et al. 2023; Renkema and Hilletofth 2022). Many of these research papers and case studies report on different aspects of SFSCs, with the *British Food Journal, Journal of Cleaner Production* and *Sustainability* prominent journals. This again indicates a concept that is attracting much research interest. These review (and supporting empirical) papers help further define and characterise SFSCs, as well as reviewing evidence and critiquing their sustainability.

Building on this past work and seeking to extend and advance the field and contribute to knowledge, this paper uses an assessment of the current state of play for SFSCs in England to do key two things. The first relates to the where question, especially the *types of farm producer* interested in SFSCs. SFSCs are often associated with smaller farms and deemed to be less interesting to larger farms. Analysis here suggests this has changed, supporting recent work by O'Neill (2024) that challenges the idea that SFSCs are largely the preserve of less intensive agricultural regions. The second contribution concerns the *state of the art foci*, particularly to recognise more explicitly infrastructure as a critical enabling condition to allow producers (large and small) interested in SFSCs to realise positive outcomes. We use these insights to scope out a research agenda for SFSCs. Specifically, we call for a sharper, holistic focus on ‘infrastructure’ as the essential means to support and provide enabling capacities to scale up SFSCs as territorial forms of rural urban food economy provisioning (Kneafsey et al. 2021; Marsden and Morley 2014; Maye et al. 2023). Reconfiguring infrastructure is often essential for transforming national, regional, and local food systems. Consequently, SFSC research should consider infrastructure as a key research avenue for analysis and policy mobilisation. From a theoretical aspect, this demands greater engagement with infrastructure-inspired scholarship (Chachra 2023) to conceptualise the transformative capacities of SFSCs and alternative food networks (AFNs) (see Goodman et al. 2012).

The next section of the paper discusses the treatment of infrastructure in the SFSC literature. In the methods section, we summarise a cross-sectional study conducted by the authors that examined SFSC opportunities and barriers in England. We then present key findings from this work as a current state of play assessment, revealing widespread interest in SFSCs amongst the farming community (across all farm sizes) combined with a noted demand for supporting infrastructures to enable farm businesses to engage in these marketing channels. The second half of the results and the discussion sections elaborate next steps for research and policy innovation and action to address infrastructure needs for local and regional food economies, including different forms of infrastructure (e.g. physical, digital) alongside human and social capital.

## Infrastructure and the SFSC literature

With hundreds of research papers now published on SFSCs, not to mention many reports, books, and book chapters commenting in some capacity on these alternative styles of food provisioning, there is no space here to review the literature in an exhaustive sense. Instead, we use key papers, particularly those that originally developed the concept combined with recent review papers (see Table 1), to build and bring to the fore, to borrow the sociological phrasing of Goffman (1978), infrastructure from the ‘backstage’ more to the ‘front stage’ in SFSC studies. We have selected key foundational SFSC papers and those that more explicitly consider infrastructure in some capacity. The list is not intended to be exhaustive or complete, reflecting instead key contributions to this debate, including review papers that give good geographical coverage and wider topic-based strategic overviews. In the analysis that follows our discussion is not limited only to papers cited in Table 1. The objective is to use Table 1 as a starting point to connect SFSC and infrastructure literatures more explicitly as a research agenda and to then build on those selected papers and expand further to show links with the wider established SFSC literature. We argue to focus less on farm to fork ‘reconnection’ at the farm or retail end of the supply chain and more on often ‘hidden’ but essential infrastructures and intermediate steps in, around and between production consumption relations. In essence, infrastructures that provide the means to provision locally and scale up AFNs. This brings in new framings for SFSCs linking with ideas such as the ‘foundational economy’ that emphasise material and social assets for local wealth building.

**Table 1:** Key cited literature on SFSCs and infrastructure

Author/s	Research design	Key findings
Marsden et al. (2000)	Qualitative survey of farmers in rural Wales. Part of the IMPACT project	Introduces the SFSC concept and three types, evidenced using interview data of farm-level strategies in rural Wales to enable farms to sell direct
Renting et al. (2003)	Quantitative survey of SFSC strategies in Europe. Part of the IMPACT project	European-level paper introducing the SFSC concept, the quality turn, and the potential for SFSC strategies to support farmer livelihoods and rural economies
Goodman (2004)	Conceptual review paper—drawing and responding to IMPACT data and papers	Paper that challenges, in part at least, the extent to which SFSCs signify a ‘paradigm change’ in agri-food systems
Ilbery and Maye (2005)	Qualitative survey of farmers in Northumberland and the Scottish Borders. Part of the SUPPLIERS project	Extends the SFSC concept by adding ‘upstream’ as well as ‘downstream’ component. Argues SFSCs are hybrid, including for infrastructure support
O’Neill (2014)	Interviews with farmers, local food businesses, consumers, and policymakers in East Yorkshire, England	Challenges the idea that local food/SFSCs only prosper in non-intensive agricultural regions. SFSCs associated mostly with smaller farms in the region
Chiffoleau et al. (2019)	Case study of open-air market in France and solidarity purchase groups in Italy	Case studies underscore the central role of social processes in supporting new economic models (SFSCs)
Chiffoleau and Dourian (2020)	Literature review to establish research agenda for SFSCs	Organised as a multidimensional approach to sustainability, the review finds common support for SFSC social benefits but mixed for economic and environmental outcomes. Health and governance remain unexplored
Krzywoszynska et al. (2022)	Visioning and backcasting process, post-COVID, with key local food businesses and organisations in the UK	Proposes a vision, models, and pathways for local food actors, including building new infrastructures (physical and habitual) to transform food systems
Renkema and Hilletofth (2022)	Systematic literature review of peer-reviewed papers on SFSCs until December 2021	Highlights the importance of ‘intermediate’ SFSCs in the food system and the crucial role intermediaries play in such arrangements
Jia et al. (2024)	Systematic review of SFSCs (92 articles) with papers from global case studies	Summarises factors driving adoption of SFSCs and challenges to their development, including lack of infrastructure and market dynamics

The SFSC concept has a lineage and connection with European rural development policy. It emerged in the early 2000s, from a European framework project that was tasked with identifying new development pathways for farmers that were trapped in industrial agriculture commodity chains and a cost-price squeeze (Marsden et al. 2000; Renting et al. 2003; van der Ploeg et al. 2000). As part of a mix of pluriactive, multifunctional strategies, SFSCs were a central pillar in a new rural development dynamic that repositioned farmers at the heart of rural development. As Goodman (2004, p.8) argues, AFN/SFSC arrangements were ‘new economic spaces’ promoted on ‘the ability of quality food products to secure premium prices’ and central to a new ‘market-led, value added model’. The different types of supply chain were stylised as three types: face-to-face, spatially proximate, and spatially extended (Marsden et al. 2000) and subsequent studies emphasised the need also to include ‘upstream’ as well as ‘downstream’ supply chain arrangements (Ilbery and Maye 2005; Ilbery et al. 2005). These studies emerged also at a time when the negative impacts from industrial agriculture became increasingly apparent, particularly in the UK coming in the aftermath of bovine spongiform encephalopathy (a cattle disease) and the Foot and Mouth crisis.

In these early studies, links with infrastructure were indeed present, particularly the relationship in livestock farming between animal production, transport, and the impact of a lack of abattoirs to process animals locally. In SFSC studies, infrastructure has arguably always had ‘co-presence’ but more in the backstage and often piecemeal. If we step into the world of public food procurement, for example, arguably the most topical way to ‘scale up’ local food chains, the role of infrastructure is ever present in discourses and practices to provide the means to deliver sustainable food (Morgan and Sonnino 2008). The right school meal contracts for example, as well as the necessary technology, cooking skills and kitchens—these are all in different ways linked to legal, material, or social infrastructures. In general, however, the focus frontstage has remained fixed at the two ends of the ‘reconnection story’, by which we mean the local farm and retail outlet. A great deal of work has focussed, for example, on the direct selling form of SFSC, in particular farmers’ markets and their capacity to ‘perform’ reconnection through social embeddedness. To a much lesser degree, work focussed on spatially proximate and spatially extended SFSCs, or what Renkema and Hilletofth (2022) term ‘intermediate SFSCs’ (Table 1). They call for future research on the roles intermediaries play in these arrangements. The role played by intermediate actors is also one of three future research priorities noted by Chiffoleau and Dourian (2020) in their excellent review. This covers actors such as food artisans, small independent businesses, chefs/restaurant owners, wholesalers and retailers, and others involved in supporting SFSCs. Work in this domain references also the importance of digital infrastructures to support online sales. Local food economies have also evolved significantly since early inceptions, with a growing body of work in urban food contexts (see Petruzzelli et al. 2023), introducing many new examples around SFSC arrangements, including food partnerships, alliances, clubs, and buying groups, signifying evolution and experimentation with the SFSC concept.

We detect this renewed energy and evolution of the concept in the recent review papers on SFSCs (Table 1), with reference to intermediary roles, but some persistent questions and topics tend to dominate the foci of many SFSC studies. One common theme is the emphasis on the values, beliefs, and motivations that inspire food actors to get involved in this type of food economy and the degree of distinctiveness from conventional or global food systems (Chiffoleau and Dourian 2020). Another dominant theme relates to assessments of supply chain practice and performance through different forms of sustainability criteria, spanning economic, social, and environmental domains and more recently health and ethics (see e.g. Evola et al. 2022; Filippini et al. 2023; Kłoczko-Gajewska et al. 2023; Reina-Usuga et al. 2023, as well as Chiffoleau and Dourian 2020). These are important questions, but they tend to dominate and arguably stifle new perspectives in the literature.

However, reference to infrastructure is present in recent SFSC papers and research reports (Table 1). Jia et al. (2024) reviewed 92 research articles, for example, assessing factors driving adoption and challenges, the latter including a lack of infrastructure, as well as information asymmetry, regulatory barriers, and market dynamics. In the infrastructure description, one study highlighted the impact of existing infrastructure not being able to meet the demands required for SFSC expansion (Ge et al. 2022). Other papers emphasise the importance of accurate data collection, efficient cost management, and forecasting as a means to enhance productivity and sales, with infrastructure necessary to support data collection, storage, and analysis (e.g. Zwart and Wertheim-Heck 2021). Studies refer also to the importance of transport and distribution systems (e.g. Zhang and Yu 2021). Paciarotti and Torregiani (2021) shine a light on the question of logistics and food distribution systems as a means to improve the effectiveness and sustainability of SFSCs, with a view to encouraging farmers to adapt an open approach to ‘innovative distribution systems’ for shorter circuits. Chiffoleau et al. (2019) examine new economic models for SFSCs—specifically an open-air market promoting SFSCs in France and a partnership arrangement between agricultural co-operatives and solidarity purchasing groups in Italy as new forms of ‘economic organisation’, each of which is underpinned by critical forms of infrastructure (material, legal, social, etc.).

Building on this work requires an overarching, more holistic research agenda that collates and evaluates specific infrastructure examples and insights to consider in a more rounded and explicit sense what constitutes ‘critical infrastructure’ assets for SFSCs. As Jia et al. (2024, p.10) argue, ‘addressing the issue of inadequate infrastructure is crucial for the successful implementation and growth of SFSCs’. A recent report, organised in the wake of the Covid-19 pandemic, included a scenario and visioning exercise conducted with UK sustainable food system stakeholders (Krzywoszynska et al. 2022). The visioning process mapped where the sector wanted to get to and how to get there to become ‘a force for good’, i.e. how local food systems and SFSCs become an engine for societal, economic, and ecological transformation. The study identifies three ‘arenas for action’, and one arena, of particular interest here, was ‘building new infrastructures’. These are actions within local food systems to construct physical, digital, and legal infrastructures to actualise the values of the local food sector. Examples include material infrastructures such as abattoirs or distribution hubs, online platforms (Open Food Network, Better Food Traders, Dynamic Procurement technology), and fair contracts and agreements (e.g. Riverford Organics, which is majority owned by its employees) as governance mechanisms and structures.

A key argument of Krzywoszynska et al. (2022, p.23) is that alternative infrastructures ‘can both compete with the dominant ones, as well as thrive in the areas omitted by the mainstream food system’. The infrastructures have functional usefulness, in the sense that they can scale up circulation of local produce and connections between actors in the local food system, but they can also ‘serve as powerful normative tools, exemplifying the kinds of livelihoods and customer experience which can be achieved through value-based market mechanisms’ (ibid., p.23). The ideas in this research are an important first step in bringing critical infrastructure more to the front stage in SFSC research or, as Chachra (2023, p.6) calls for, to bring it more ‘to our conscious attention’. This sits too alongside work which usefully challenges assumptions that associate SFSC activities with certain farming styles, food systems, places, and regions (O’Neill, 2014), e.g. more the demise of smaller farms, less marginal production systems, and targeting speciality niche markets. The next step, which the rest of this paper elaborates, is to scope out the agenda, building on the current state of play.

## Research design and methods

In this methods section, we explain the iterative nature of the work, informed by multiple data sources (see Table 2). As noted above, this paper is an intervention that calls for greater attention and sets an agenda for more research on infrastructure to support SFSCs. To build this case, we draw from findings from a recent 2023–2024 study supported by the National Innovation Centre for Rural Enterprise that examined SFSCs in the UK. The project comprised a survey of farmers in three regions of England, a series of interviews with SFSC stakeholders in England, and a participatory workshop to co-develop ideas, priorities, and actions around the idea of infrastructure support for SFSCs. The paper draws on insights from all three elements to detail the current state of play and, using that, to then build a case to develop the research agenda. We summarise below the design, rationale, and procedures for each phase.

**Table 2:** Research phases and cross-sectional dataset

Research phases	Methods description and dataset
Phase 1: Farm survey	586 farms surveyed in three regions of England (see Table 3). Farm survey designed to understand the cost of doing business in 2023 and farming practices, including marketing channels. The survey was conducted between May and August 2023 and took 15–20 minutes to complete
Phase 2: Semi-structured interviews	29 interviews (all audio recorded; one hour approx. per interview) with participants based in England. Purposive sample, including SFSC operators, advisory organisations that support local food economies and three academics actively researching in this area
Phase 3: Participatory workshop	Workshop with 25 participants in February 2024, all of whom were involved in developing SFSCs, including academics, think tanks, local government organisations, and practitioners involved in SFSC businesses. The focus was on developing practical strategies to develop the infrastructure for SFSCs

The survey component was part of a larger farm business survey designed to examine farm performance and environmental management, looking in detail at marketing channels and value added, plus climate adaptation readiness. In total, 586 farms across three English regions (North East, West Midlands, South West) were surveyed, via telephone, by a commercial market research company (Table 3). The majority were livestock farms (344 farms). Crop farms and mixed farms are also present in the sample but to a lesser extent than would be the case for the whole of England. Measured by land area, most farms sampled fall into what we defined as ‘large’ farms (more than 100 hectares), with 357 farms in total. The North East has the highest number of large farms, followed by the West Midlands and the South West regions. Small farms, defined here as less than 20 hectares, are the least represented farms in the sample. For the supply chain analysis, farm businesses were asked a series of questions to ascertain the marketing channels used to sell their produce, including the relative proportion using direct and proximate selling, as well as willingness, for those not selling through SFSCs currently, do so in future, and related barriers and opportunities.

**Table 3:** Farm survey sample (three English regions and all sample)

	North East	West Midlands	South West	All sample
*Land size (ha)*				
Small—less than 20	8	28	25	61
Medium—20 to 100	46	53	63	162
Large—more than 100	129	117	111	357
Total	183	198	199	580
*Farm type*				
Livestock	108	108	128	344
Crop	43	66	47	156
Mixed	31	21	14	66
Others	3	5	12	20
Total	185	200	201	586

The total sample is 586; 580 of the 586 farms surveyed recorded their land size

Alongside the survey element, which considered practices at the farm level, a second phase utilised semi-structured interviews to capture informed explanations from those with first-hand experience of operating or researching short food supply chains (Table 2). Purposeful sampling, selecting interviews most able to provide insights into the research questions (Patton 2015), guided the identification of potential interviewees. The interview schedule contained five sections: background of the interviewee and SFSC involvement; defining features of SFSCs; infrastructure requirements for SFSCs; evidence needs and policy support; and final reflections. Infrastructure requirements received the most attention given the purpose of the research. This section included questions relating to: the importance of infrastructure for SFSCs, what types of infrastructure are necessary for the effective operation of SFSCs, as well as soliciting examples of successful and unsuccessful SFSCs and reflection on the degree to which outcomes related to infrastructure and why.

In total, 29 interviews were conducted, with participants based in England. Interviewees included SFSC operators in a range of product chains (e.g. meat, seafood), formats (e.g. farmers’ markets, direct sales), scales of operation (i.e. local, regional, national), and organisations (e.g. private sector, local authorities, social enterprise). In addition to SFSC operators and advisors, three academics with substantial track records in SFSC research were interviewed. The purposeful sample thus captured deliberately the diversity of SFSCs, and related expertise and engagement, in England. Interviews occurred between spring and autumn 2023, mostly using video conferencing software (e.g. Zoom). Data analysis followed the approach of Braun and Clarke (2006). First, members of the research team independently undertook *NVivo* coding of interview transcripts to generate first-order codes that reflected the language and meaning of interviewees. Team members then discussed the codes generated to agree a common list of codes and definitions, before coding all interviews according to the agreed coding framework. Subsequently, axial coding occurred, identifying relationships between codes and second-order themes agreed to by the research team.

The third phase comprised a participatory workshop with stakeholders, which took place in person during February 2024 (Table 2). In total, 25 participants took part, from a range of backgrounds, all of whom were actively involved in developing SFSCs, including academics, think tanks, local government organisations, and practitioners involved directly in SFSC businesses. The workshop had two main elements. The first part focussed on evidence and knowledge exchange with presentations and discussion of recent research related to SFSCs, including a summary of key findings from the work generated from Phases 1 and 2 described above. The second part then invited participants to discuss infrastructure examples and types presented, to explore the key barriers and opportunities for developing infrastructure, focussing on the next 3–5 years, including practical actions.

## Results

In the analysis that follows, we use key insights from across the three research phases to detail the current state of play for SFSCs in England and build a case for greater attention towards infrastructure as a means to support SFSCs. Specifically, we present key findings from the different elements to explain why we need greater focus on infrastructure for SFSCs. The farm data, for example, is interesting from a commercial agriculture and scaling up perspective, with the interview and workshop data adding critical insights in relation to how SFSCs benefit local economies and what types we should consider.

### Assessing the current state of play in England: farm business and stakeholder perspectives

#### Farmers’ marketing channels, local SFSC preferences, and infrastructure needs

**Fig. 3:**
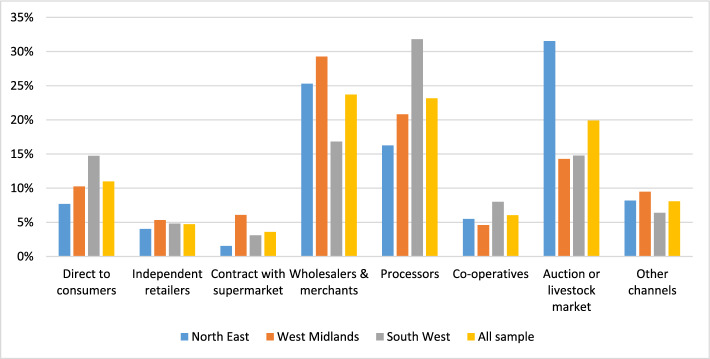
Farm sales, by value, to different marketing channels, by region and for the sample overall (Source: authors’ own survey and completed May–August, 2023)

Based on our survey evidence, Fig. 3 provides a breakdown of total farm sales, by value, via the main marketing channels. The percentages indicate the average share of sales by farms per sales channel, detailing the distribution across different channels by the three regions and the sample overall. If we look at the ‘all sample’ figures, for example, we see that traditional marketing channels are important for farm businesses: with wholesalers and merchants accounting for 24% of sales, while processors, and auctions and livestock markets, accounting for 23% and 20%, respectively. Sales to co-operatives (6%) and contract arrangements with supermarkets (4%) are less significant, although intermediate channels may well sell on to supermarkets. Direct sales, which includes pick your own, own farm sales (face-to-face and online), farmers’ markets, farm shops, and box schemes account for 11% overall, as an average share of sales by farms in the sample. ‘Other’ channels, which covers, for example, farms selling to other farms, online or private use at home, are quite small.

Sales by region are quite similar. Farm size data are more revealing, with direct-to-consumer sales more concentrated amongst smaller farms (57%). ‘Smaller’ farms could include some big businesses, particularly poultry or pig farms (no land but indoor rearing). Larger farms sell more into wholesale and processing channels (57% combined). The majority (76%) in the sample reported no direct sales. These direct sales figures are consistent with the Farm Business Survey for England data (Defra 2023), which reported that 10% of farms (of 52,500 surveyed) undertook their own processing and/or direct sales of produce in 2020/2021. Sustain and RSPB’s (2021) survey also reported similar figures with 86% of farms supplying to supermarkets, processors, and manufacturers, and with only 12% selling directly to final consumers (Woodward and Hird 2021).

Survey respondents reported whether they would like to increase the share of their sales accounted for by direct or local sales channels. Over a quarter expressed an interest in each of our three farm types, with 29% of livestock farms, 43% of crop farms and 36% of mixed farms responding positively. Similarly, positive responses were elicited from around a third of respondents in all three regions. The findings are similar to those reported by Woodward and Hird (2021), who found that 36% of farms in their survey would prefer to sell more products directly to consumers (the average of the three values in our study is 34%). We also see differences, particularly between livestock and crop farms, with the latter most positive towards selling more locally. Responses by farm size (measured in hectares) show a higher proportion of large farms would like to increase their direct/local sales (37% compared to 26% small and 28% medium). In the literature, SFSCs are generally associated with smaller farms and producers, and regarded as less interesting to larger ones (see O'Neill 2024, 2014). The survey results suggest that this association no longer holds—we witness engagement and widening interest in SFSCs amongst larger-scale farmers. Different factors may be influencing these responses. Large farms are least likely to sell locally at present, which may explain why they are keener to increase local sales. Smaller farms already sell more locally, so may not perceive a need or have little scope to do more. During the Covid-19 pandemic, local and direct sales performed well, so these data may reflect also renewed interest in local food and SFSCs.

**Fig. 4:**
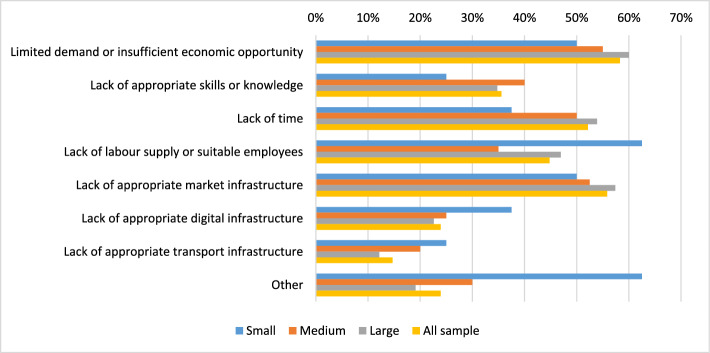
Main barriers to increasing farm sales via local/direct markets by size in hectares and for the sample overall (Source: authors’ own survey and completed May–August, 2023)

Overall, we find positive engagement and willingness to engage in SFSCs. This is not always the case, as Enthoven et al. (2023) reported in their study of fresh vegetable farmers’ decisions to participate in such channels, particularly if it involves selling large shares of their produce through such channels. In the findings reported here, several barriers for farmers were noted also for those not interested in increasing farm sales via direct or local marketing channels (Fig. 4). Smaller farms indicated a lack of time and labour as key barriers, compared to medium and larger farm enterprises that highlighted a skills and knowledge gap, given that larger farms have less existing direct experience of SFSCs. Importantly, a significant proportion cited infrastructure issues as barriers, particularly market infrastructure (Fig. 4)—see also Woodward and Hird (2021).

#### Stakeholders’ perspectives on SFSC benefits and making links with infrastructure

The farm survey findings indicate that interest in SFSCs is widening but several barriers or conditions need to be overcome to enable producers of different sizes to realise positive outcomes from SFSC market channels. As well as farmer perspectives, the following section assesses the current state of play from stakeholders with a strategic oversight of SFSCs in England, including underlying attributes and priorities. This analysis predominantly draws on evidence from the interviews. In the SFSC literature, ‘shortness’ is defined not as minimising distance per se but more the flow of information from producers to consumers and the intention to reduce, where possible, intermediate links (Ilbery and Maye 2005; Marsden et al. 2000). Both attributes were evident in the stakeholder interviews. Discussion concerning the number of intermediaries was the most popular way that participants identify short chain features. Another striking feature is the way stakeholders expressed ‘an economy of values over monetary value’. Interviewees described this in terms of ‘access to good food’, the idea of a ‘better food system’, and SFSCs as part of ‘inclusive systems’. For instance, one interviewee expressed this through the idea of fairness:

‘…fair to the workers, fair to the farmers, fair to the animals … it would be a set of attributes and that could include chocolate from the Ivory Coast. The shortness of the supply chain is a reflection of the relationship as opposed to the transactional approach’ [F8].

During the interviews, discussions switched from capturing specific ideas about SFSCs (reducing intermediate steps in the supply chain) to the values which should underpin food systems. The local economy is an enabler of multiple outcomes and, as one interviewee expressed, trying to ‘get more systemic thinking around looking at economy, environment, health and social issues related to food and farming collectively rather than in silos’ [F23]. In these initial discussions, infrastructure is often not front stage but the need to transform the food system certainly is. On the producer side, discussion concerns what makes SFSCs attractive, particularly to smaller farmers, especially ‘the inequity in the bargaining position of smaller farmers’ [F13] outside SFSC models. Here ‘infrastructure’—as new modes of organising—is more ‘present’, especially around ideas such as aggregator hubs, which, as one interviewee argued, are ‘not producing themselves, but they’re representing small-scale food producers within their region and aggregating … and representing local farmers, and handling the packaging and distribution and customer services on their behalf’ [F20]. This includes also ‘hubs’ and ‘farm-based hubs’, such as Lake District Farmers. Food hubs in England range from 20 customers up to 12-14,000 customers.

During the interviews, several interview participants (e.g. F6, F14, F15, F16, F18) also identified rural–urban connections as a productive way to consider SFSCs purposes, and their capacity to help rural economies to connect with urban areas, with infrastructure an essential part of making this happen. Others recognised that given the evolution of SFSC models in recent years, the emphasis is both on rural and urban economies and the generative potential to think more in terms of rural–urban linkages and territorial food systems.

In the interviews, we decided not to ask direct questions about the benefits of SFSCs (mostly to save time and keep focus) but discussions around why supporting infrastructure was important often led to comments regarding the benefits this would generate. The interviews covered a range of themes and examples, but most of the discussion focussed on improving producer returns, issues of power and the ability to control the supply chain. In a sense, this is not so new, given this was a key rationale when SFSCs were first proposed, but the discussions related more directly to infrastructure for its enabling capacity. For example, key motivations related to improving the price share for farmers and producers (F11, F3), increasing bargaining power (F13), using food hubs and digital platforms to facilitate local food economies (F20, F3), and implementing infrastructure to give farmers greater control and to ensure their survival (F24, F25). This is particularly evident in livestock, with many farm businesses already working at a loss and given reduced subsidy payments post-Brexit, as well as the importance of generating and returning value added to farmers. Abattoirs and food hubs feature strongly in this context.

Interviewees also made important observations regarding using infrastructure and SFSCs as ‘sites for experimentation’. Farmers’ markets, in particular, were considered an excellent place for this, with relatively low set-up costs, where producers can ‘essentially test or refine your business model and your role in that model really flexibly’ (F23). Other models, not yet applied in England, were also noted, such as shared processing incubators, which have been applied in the USA and again are a means to expose a business to ‘gradual expansion … and manage that risk to financing and the need for financing for micro businesses that are becoming small businesses’ (F23). Interviewees referred also to environmental benefits as part of infrastructure design, particularly linked to the climate emergency, carbon emissions, and carbon footprints (e.g. F19, F2). As one interviewee insisted, the emphasis of infrastructure design should also be to solve more than one crisis.

A final theme concerned infrastructure design to support SFSCs as a means to enable wider human and social capital benefits, including community engagement. This was expressed in different ways, including the capacity for SFSC businesses to bring ‘a sense of place’ (F19), and to initiate training, business support, and networking opportunities to enable people to do peer-to-peer learning (F2). This includes shops, food hubs, markets, market stalls, box schemes, and wholesalers, with some performing multiple roles. As the interviewee explained, this concerns ‘relationships rather than just transactions … it’s really about selling food they know who’s grown it, they know who’s produced it, and there’s a relationship there in buying and selling and that in turn means that fair prices are paid, and the balance of power is equal rather than completely unequal’ (F2).

### Infrastructure support for SFSCs: using stakeholder insights to build a research agenda

#### Infrastructure’s role and importance

The section above reports how stakeholders almost intuitively connect SFSC benefits and purposes to infrastructure, sometimes without conscious acknowledgement. It might be obvious and clearly part of what is needed, but it also shows infrastructure’s ‘backstage presence’ not only in the SFSC literature but also in practitioner ontologies. In this section, analysis from the interviews and workshop is more explicit about why infrastructure is important, what it means, and how we express, organise, and communicate priority actions.

We found strong consensus in the interviews and the workshop regarding the importance of infrastructure for SFSCs. We see also qualifying specificities in relation to activities or sectors. This is not surprising given the heterogeneity of actors, emphasising a need to tailor interventions. On the importance of infrastructure, interviewees commented, for example, that ‘it’s a deal breaker … One of the key reasons that supermarkets dominate so much is that there is not the infrastructure to support small, small supply chains’ (F11). Described in relation to enabling access to markets, another interviewee underscored digital infrastructure as ‘vitally important … for local food economies’, including continued investment, given the wider food retail environment and competition from supermarkets as well as online retailers and social media platforms such as Amazon, eBay, Uber, or Facebook. In their words, ‘people are just so accustomed to being able to just get what they want by pressing a few buttons on their phone’ (F20).

Referring to meat and dairy, one interviewee reflected on the centralised nature of food economies, creating what they called a ‘zero line of sight’ scenario, meaning the nature and role of infrastructure have become hidden. Several interviewees also noted this; one interviewee stated that the question of infrastructure does not receive enough emphasis, often because ‘people don’t see it’ (F15). It is also clear from the interviews that fixing infrastructure alone will not solve the problem. As one interviewee put it, ‘there’s a whole constellation of factors, unfortunately, and then a lot of it’s down to luck and timing’ (F21).

Recognising the overall importance of infrastructure, and the general lack of emphasis on its role, interviewees noted that discussions were starting to occur more recently. In the early development of local food economies emphasis was mostly towards the retail outlets—farm shops, farmers’ markets, etc. However, in England infrastructure has become more prominent in policy discussions in the last four or five years. This is thanks to discussions and workshops held at major events such as the Real Oxford Farming Conference, the Oxford Farming Conference and Groundswell, and pioneering campaigning work led by the likes of Sustain and the Sustainable Food Trust (e.g. Lawes-Johnson and Woodward 2022; Sustain and RSPB 2021).

It is interesting to unpack how interviewees responded to the general prompt about the role of infrastructure for SFSCs. Remarks are often quite specific, relating to infrastructure types, particular SFSCs, or agri-food sectors. Interviewees referred, for example, to ‘digital infrastructure’ (F15, F20), as well as ‘informational infrastructure’, access to data, and data and data providers (F22). The same interviewee also described access to staff and the inability to source staff (human capital) as a major barrier (F22). Participants referred to inspiring cases, currently operating in England, to support their argument. This included the Bath and North East Somerset model for dynamic public procurement (F15); Better Food Shed, a not-for-profit wholesaler (F25, F2); Pipers Farm which supports hundreds of local farms in Devon and Cornwall to sell locally (F11); Ooooby’s vegetable box platform for small farmers (F3); the Open Food Network and the sharing infrastructure it provides (F8); and the Organic Farmers’ Network, a cooperative model (F19).

#### Priority topics and action arenas

Three key themes relating to SFSC infrastructure stand out as priority topics. The first is discussion around small and mobile abattoirs. This topic has received considerable attention and discussion in recent years—it is not a new challenge but has recently intensified as the number of registered abattoirs in England has fallen from circa 25,000 in the 1930s to 147 by 2023 (AHDB 2024; APGAW 2020). Interviewees reiterated the need for small abattoirs to support livestock-based SFSCs, and the fact that required infrastructure continues to decline (F14). As one interviewee put it, ‘the amount of concern I get about the state of the local abattoir network is incredible. I get far more people expressing concern about that than I do about their support payments going. The rate at which they’re disappearing is horrendous’ (F13). Interviewees also considered the case for mobile abattoirs, which were deemed to be one practical solution, but recognised that they also had logistical and costs challenges, including requiring adequate chiller and farm storage space (F16, F3), reiterating the need for wider material infrastructures for these interventions to be viable.

A second theme relates to food hubs and wholesalers. Some interviewees regarded food hubs as ‘top of the list’ in terms of priority actions for infrastructure. This is supported by earlier analysis—a farm survey run by Sustain in 2021, for example, noted food hubs and dairy processing as priority interventions (Woodward and Hird 2021). Some interviewees were more critical, however, suggesting ‘regional food hubs had been attempted but had not worked’ (F22). Some participants in the workshop were also dismissive of the priority given to food hubs, regarding it as more of ‘an academic agenda than actual need or solution’. Of particular interest is an emerging discussion about the role of food hubs and wholesalers. One interviewee, in particular, was adamant that it should not be either or and that established wholesalers also have a critical role to play in supporting local food economies (F6), stressing the advantages of integrating wholesalers that combine long and short channels.

The third topic relates to public procurement. Regarding this topic, interviewees noted several infrastructure challenges, namely: the ability to secure enough supply locally (F10), ensuring a consistency of supply for hospitality (F19), better data collaborations (F25), and information on what farms are producing for local authorities (F15). For dynamic procurement, infrastructure is essential to connect regional production (agroecological farms), regional processing, regional distribution (existing SMEs), and regional buyers (supported by a compliant procurement framework). In this model, dynamic automation technology brings operational efficiency and opens new market access opportunities, but it needs the right level of national and regional support.

## Discussion

This paper calls for explicit attention to the role of infrastructure in supporting SFSCs as transformation pathways for food systems. The discussion section uses the material presented so far as the basis for presenting a research agenda to advance future work in this space. As explained above, the motivation for this intervention was inspired by two main sources. The first is a review of recent SFSC research—here we find a dramatic increase in terms of published papers, projects, and policy documents, including at a European and international level (Chiffoleau and Dourian 2020; Jia et al. 2024). This literature mentions infrastructure needs in a few instances, but it tends to be sporadic or as we call it more ‘backstage’. The second is more empirical, based on findings from our farm business survey, which revealed widespread interest in SFSCs as marketing options for farms in England, to a much greater degree than anticipated, particularly within the crop sector and larger farms. Neither are usually associated with SFSCs (cf., O’Neill, 2014, 2024). Infrastructure is perceived as a major barrier to access such markets. The argument is therefore that if we aim to scale up SFSCs, particularly to make them appealing to larger, commercial farms and commodity chains, addressing infrastructure becomes an urgent imperative. However, as we also see through the interview and workshop data presented, this requires further analysis and granularity, which we elaborate below as the basis for a future research agenda. In what follows, we identify key points to guide this work.

Firstly, we identify a strong and clear consensus from practitioners about the need to focus more explicitly on infrastructure support for SFSCs, with the caveat that this requires specificity, contextualisation, and avoidance of silver bullet messaging. Workshop participants emphasised infrastructure in relation to wider systemic challenges, for example, how food is valued, which implies that infrastructure per se is not the problem, but more the wider governance of the food system. This supports other workshop comments about the way government has effectively devolved food security to supermarkets and the food industry. Fixing infrastructure alone will not resolve the problem. We should avoid, then, an overly simplistic idea that infrastructure alone will fix food systems, pushing it instead as a critical ‘front stage’ priority action for SFSC development, but alongside wider alliance building (between local food and health and community well-being, etc.) and in combination with other efforts to improve evidence building, data gathering, and indicators for impact.

Linked closely to the first consideration, the second point is recognition that this is not an academic agenda. What we propose and find here represents and reiterates points noted in several excellent industry reports (e.g. Lawes-Johnson and Woodward 2022; Woodward and Hird 2021), representing in a UK context work by Sustain and other sustainable food organisations. This emphasises the need for co-design in ongoing future research also, as was the case with the participatory workshop for this piece. As stakeholders at that workshop noted, what is helpful is to co-develop a more holistic understanding and language that describes infrastructure types and their combinations for different sectors, supply chains, and regions. As introduced in this paper, the framework proposed by Krzywoszynska et al. (2022) is a useful starting point, identifying for example material, virtual, and legal infrastructures. Workshop participants were asked to comment on these and other examples and many could relate, although some struggled to articulate legal forms, even though they are essential if we consider, for example, contracts that determine payment procedures, worker conditions, etc. More work is needed to develop appropriate typologies, taxonomies, and vocabularies to describe different infrastructure types, how they work in combination with one another in different contexts and alongside human and social capitals.

Having suggested above that the research agenda for infrastructure for SFSCs be co-designed and transdisciplinary, a further research priority, then, is to link this agenda to wider bodies of social theory. Specifically, new work on infrastructural systems (Chachra 2023) and work in regional economy, place-based policy, and diverse and alternative economies is valuable, including work on doughnut economies (Raworth 2018), foundational economies, and well-being (Froud et al. 2020, 2022; Heslop et al. 2019). This not only injects new social and economic theory into SFSC studies but more critically provides alliances with wider scholarship which also recognises and calls for greater attention to infrastructure and support intermediaries as essential nodes to maintain and support ‘everyday social life’. Such thinking also strengthens local rural urban interlinkages, via demand and supply-side perspectives. Of particular relevance for SFSCs is the supply-side argument, which in England to date has orientated around anchor institutes and public procurement (the Preston Model) but it offers a novel way to conceptualise infrastructure for SFSCs more generally.

The final point is more specific, in terms of priority research actions on the ground, particularly related to place-based strategies and key arenas of action linked to public procurement, food hubs, small abattoirs, and the provision, where possible, to share SFSC data in open science frameworks. This highlights the need for differentiation in relation to SFSC needs. As one interviewee argued, ‘when you’re thinking about infrastructure and operations, they are very, very different and very specific depending on what product or blend of products that you’re selling’ (F19). Other interviewees made interesting remarks regarding what sectors and infrastructure deserve further consideration. They suggested, for instance, that arguments about meat and abattoirs and the horticulture sector are very important but also now quite well made. Attention thus needs to widen to other sectors, particularly oilseeds, grains, and protein crops as sectors that need greater emphasis and support in both their rotations and new markets. Infrastructure needs also vary from region to region, which means considering geographical areas in terms of needs.

## Conclusion

*
‘Humans are disturbingly good at filtering out anything in our vision that we’re either accustomed to seeing or which doesn’t appear meaningful…Between their familiarity and their unannounced, unexplained presence, infrastructural systems are easy to see but just as easy to ignore, unless we bring our conscious attention to bear on them’ (*Chachra 2023*, p.6).*

This paper argues that greater focus on infrastructure to support and enable SFSCs is an important next step for research. This is crucial to build resilience capacity in agri-food economies and to enable producers interested in SFSCs to realise positive outcomes from these modes of food provisioning. The analysis of the current state of play in England supports this both in the sense that farmers of all sizes are more engaged but see infrastructure as a major barrier, echoing the views of other stakeholders. The agility of more local food economies, combined with the power of virtual forms of infrastructure to connect producers and consumers, was notable during the Covid-19 pandemic. As reiterated in several stakeholder interviews, the pandemic highlighted, albeit fleetingly, SFSCs’ resilience capacities and the need to build infrastructures to support food systems in the future. Despite these lessons, and a recent burgeoning of research on SFSCs in Europe and internationally, the role and importance of infrastructure to support SFSCs arrangements remain piecemeal and too often ‘backstage’. In conclusion, future research needs to bring infrastructure ‘front stage’, echoing Chachra’s (2023) argument above, meaning an urgency to consider infrastructure in a more meaningful holistic sense as a key avenue for analysis and policy mobilisation in England and the devolved nations of the UK and translatable also to market economies in wider international contexts. Key steps for a future research agenda include developing a common understanding of infrastructure types and combinations for sectors. As well as a consensus for action, we have here then tentative steps for a future research agenda.

## Data Availability

Quantitative and qualitative data underpinning this study are available in anonymised formats upon request. Please contact the corresponding author.

## Abbreviations

AFNs: Alternative food networks
SFSCs: Short food supply chains
SMEs: Small and medium enterprises
